# Late UP-state Bias in Cortico-amygdalar Coordination

**DOI:** 10.1523/JNEUROSCI.0449-26.2026

**Published:** 2026-10-06

**Authors:** Yu Sato, Tetsuhiko Kashima, Shota Morikawa, Haruki Takeuchi, Yuji Ikegaya

**Affiliations:** ^1^Graduate School of Pharmaceutical Sciences, The University of Tokyo, Bunkyo-ku, Tokyo 113-0033, Japan; ^2^Graduate School of Sciences, The University of Tokyo, Bunkyo-ku, Tokyo 113-0033, Japan; ^3^Institute for AI and Beyond, The University of Tokyo, Bunkyo-ku, Tokyo 113-0033, Japan; ^4^Center for Information and Neural Networks, National Institute of Information and Communications Technology, Suita City, Osaka 565-0871, Japan

**Keywords:** basolateral amygdala, medial prefrontal cortex, phase-dependent activity, slow oscillation, UP state

## Abstract

Neocortical slow oscillations temporally organize neuronal activity across distributed brain regions, but the coordination of basolateral amygdala (BLA) activity with these oscillations remains unclear. We investigated BLA neuronal activity relative to neocortical slow oscillations in male mice, primarily under urethane anesthesia. These oscillations are characterized by rhythmic alternation between active (UP) and inactive (DOWN) states at 0.3–3 Hz. Using unit recordings and calcium imaging, we found that BLA neuronal activity was sparse but preferentially associated with the later phase of the neocortical UP state, while calcium activity showed local spatial organization with greater synchronization among nearby neurons. Medial prefrontal cortical neurons projecting to the BLA were more likely to continue firing into the late phase of the UP state than other prefrontal neurons. Deep whole-cell recordings revealed transient depolarizations in BLA neurons preferentially during the late phase of UP states. Both the probability and timing of these depolarizations varied with UP-state duration, and voltage-clamp recordings revealed temporally anticorrelated changes in excitatory and inhibitory conductances. Thus, these findings define a temporally structured and probabilistic mode of neocortex–BLA coordination during slow oscillations.

## Significance Statement

Neocortical slow oscillations organize long-range neuronal coordination during sleep-like states, but their temporal relationship with basolateral amygdala (BLA) activity remains poorly understood. We show that BLA neuronal activity is sparse and probabilistically biased toward the late phase of neocortical UP states rather than uniformly distributed throughout the UP period. BLA activity is further characterized by temporally anticorrelated changes in excitatory and inhibitory conductances and locally organized calcium activity. In addition, BLA-projecting medial prefrontal cortical neurons are more likely to continue firing into the late phase of UP states. These results establish a physiological framework for understanding the temporal organization of neocortex–BLA coordination during slow oscillatory brain states.

## Introduction

Neocortical slow oscillations are prominent during non-rapid eye movement (non-REM) sleep and anesthesia and are observed across the neocortex of rodents, felines, and humans (Petersen et al., 2003; Massimini et al., 2004; Volgushev et al., 2006; Luczak et al., 2007). These oscillations consist of alternating depolarized (UP) and hyperpolarized (DOWN) states (Steriade et al., 1993a,b,c). By periodically modulating neuronal excitability, slow oscillations temporally organize neuronal activity not only within the neocortex but also across connected cortical and subcortical structures (Battaglia et al., 2004; Hahn et al., 2006; Isomura et al., 2006).

The basolateral amygdala (BLA) is a major limbic structure involved in diverse affective, motivational, and mnemonic processes (Janak and Tye, 2015). It receives extensive cortical inputs, including direct input from the medial prefrontal cortex (mPFC), and participates in reciprocal interactions with neocortical networks (Little and Carter, 2013; McGarry and Carter, 2017). However, the temporal relationship between BLA neuronal activity and neocortical slow oscillations remains poorly understood.

In this study, we examined the phase-dependent relationship between neocortical slow oscillations and BLA neuronal activity. We also compared the UP-state firing patterns of BLA-projecting and other mPFC neurons to assess whether the BLA receives temporally biased cortical input. Our findings reveal four main features of neocortex–BLA coordination during slow oscillations. First, BLA neuronal firing, calcium events, and membrane potential depolarizations are sparse and show a bias toward the late phase of the neocortical UP state. Second, BLA membrane potential dynamics are associated with temporally anticorrelated changes in excitatory and inhibitory (E/I) conductances, in contrast to the more balanced E/I pattern observed in the neocortex. Third, BLA calcium activity shows local spatial organization, with nearby neurons exhibiting more strongly synchronized activity. Fourth, BLA-projecting mPFC neurons are more likely than other mPFC neurons to continue firing into the late phase of the UP state. These results collectively demonstrate a temporally structured and probabilistic mode of neocortex–BLA coordination during slow oscillatory brain states. By defining the temporal architecture of neocortex–BLA interactions, this study provides insight into the physiological mechanisms that may support BLA-dependent neural processing.

## Materials and Methods

### Animals

Male ICR mice [postnatal days (P) 28–42 at the time of the initial surgical procedure; Japan SLC] were used, and all surgical procedures were initiated within this age range. Animal experiments were approved by the Animal Experiment Ethics Committee at the University of Tokyo (approval numbers: P29-9, P4-2) and conducted in accordance with the University of Tokyo guidelines for the care and use of laboratory animals. All procedures complied with the Fundamental Guidelines for Proper Conduct of Animal Experiment and Related Activities in Academic Research Institutions (Ministry of Education, Culture, Sports, Science and Technology, Notice No. 71 of 2006), the Standards for Breeding and Housing of and Pain Alleviation for Experimental Animals (Ministry of the Environment, Notice No. 88 of 2006), and the Guidelines on the Method of Animal Disposal (Prime Minister’s Office, Notice No. 40 of 1995). Mice were housed under a 12 h light/dark cycle (lights on from 7:00 to 19:00) at 22 ± 1°C, with *ad libitum* access to food and water.

### Virus injection

Mice were anesthetized with 1.5–3% isoflurane and placed in a stereotaxic frame (SR-6M-HT). Anesthesia was verified by the absence of paw withdrawal, whisker movement, and eyeblink reflexes. To confirm the preservation of mPFC→BLA projections after the tissue-boring procedure, an adeno-associated virus (AAV_DJ_-hSyn-Synaptophysin-mCherry; 5.56 × 10^13^ genome copies/ml) was injected into the mPFC (AP: 1.5 mm anterior to bregma, ML: 0.3 mm lateral from the sagittal suture, DV: 1.3 mm ventral to the dura) at a rate of 100 nl/min using glass pipettes. For imaging, AAV_dj_-CaMKII-jGCaMP7f (9.5 × 10^12^ genome copies/ml) was injected into the BLA (AP: 1.8 mm posterior to bregma, ML: 3.4 mm, DV: 4.1–4.2 mm). For opto-tagging experiments, AAV-CAG-hChR2-H134R-tdTomato (a gift from Karel Svoboda, Addgene viral prep # 28017-AAVrg) was injected into the BLA (AP: 1.6 mm posterior to bregma, ML: 3.3 mm, DV: 4.2 mm).

### Unit and LFP recording

For acute extracellular recordings, mice were anesthetized with urethane (2.25 g/kg, intraperitoneal), and anesthesia was verified by the absence of paw withdrawal, whisker movement, and eyeblink reflexes. The scalp was removed, and the animal was positioned in a stereotaxic frame, where a screw placed over the cerebellum served as a common reference and ground in all preparations. For acute BLA recordings, a single-shank silicon probe (A1x32-6mm-100-177, NeuroNexus), coated with DiO, was inserted into the BLA (AP: 1.8 mm posterior to bregma; ML: 3.4 mm lateral from the sagittal suture; DV: 4.5 mm ventral to the dura). Neocortical LFPs were simultaneously recorded from a tungsten electrode (UEWMGCSEKNNM, FHC), coated with DiI (AP: 0.4–2.5 mm anterior to bregma; ML: 0.1–1.0 mm; DV: 1.5–2.7 mm). For acute mPFC recordings combined with optogenetic stimulation, a 32-channel silicon probe with an integrated optical fiber (Buzsaki32-OA32LP-v2, NeuroNexus), coated with DiO, was inserted into the mPFC (AP: 1.8 mm anterior to bregma; ML: 0.2 mm; DV: 1.5 mm). For these experiments, an adeno-associated virus was injected into the BLA 2–4 weeks before recording. Blue laser light (473 nm; Lucir) was delivered via patch cords connected to a ferrule (PlexBright, Plexon). Light pulse trains (1 or 5 ms, 5 Hz) were delivered for 1 s every 300 s or continuously for 40 s, totaling 100–400 pulses, either at the beginning of or during the recording session. For chronic BLA recordings, mice were anesthetized with isoflurane and implanted with silicon probes (Buzsaki32-H32 or Buzsaki64L-H64LP, NeuroNexus), coated with DiO, mounted on a custom-built microdrive targeting the BLA. A frontal cortical screw electrode (AP: 2.5 mm anterior to bregma; ML: 0.5 mm) was used to record neocortical LFPs, and electromyogram (EMG) signals were recorded using fluororesin-insulated wires (AS633, Sensortechnos) inserted into the neck muscles. Craniotomies were sealed with white petrolatum, and a copper mesh cage was used for electromagnetic shielding. Neural signals were recorded after 1–7 d of recovery in the home cage. Electrode placement was verified post hoc by visualization of DiO- or DiI-labeled tracks. All signals were sampled at 30 kHz using the Open Ephys acquisition system (Siegle et al., 2017).

### Calcium imaging and LFP recording

Following viral injection, a GRIN lens (1050-002183, Inscopix) was implanted above the BLA (AP: 1.8 mm posterior to bregma, ML: 3.3–3.4 mm, DV: 4.0–4.2 mm; Resendez et al., 2016). A screw electrode for neocortical LFP recording was also implanted (AP: 2.5 mm anterior to bregma; ML: 0.5 mm). The lens was secured using adhesive (Super-Bond C&B; Sun Medical) and dental cement (Provinice, Shofu) mixed with carbon powder to prevent light leakage. A silicone impression material (Dent Silicone V, Shofu) protected the lens during home-cage activity. Two weeks later, a head plate was attached under isoflurane anesthesia. Mice were habituated to a custom-made treadmill and head-mounted stage (Nikon) for 1 h per day beginning 1 week before imaging. Four weeks after viral injection, Ca^2+^ imaging was performed using a spinning-disk confocal microscope (Ishikawa et al., 2020) at 604 × 444 pixels and a 20 Hz frame rate. Excitation was delivered using a 50 mW LED (473 nm). LFP signals were amplified with a DAM80 AC differential amplifier (World Precision Instruments) and digitized at 20 kHz via a Digidata 1440A digitizer, controlled by pCLAMP 10.7 software (Molecular Devices). The camera shutter signal was recorded simultaneously with the LFP. The timing of each imaging frame was determined from the rising edge of the shutter signal, and the corresponding slow-oscillation phase was used to align calcium activity to the LFP.

### In vivo patch-clamp and LFP recording

In vivo whole-cell recordings were performed as described previously (Sato et al., 2021; Noguchi et al., 2022; Samejima et al., 2023; Mizuno and Ikegaya, 2024). Mice were anesthetized with urethane (2.25 g/kg, i.p.), and anesthesia was verified by the absence of paw withdrawal, whisker movement, and eyeblink reflexes. For BLA recordings, a 1.8-mm-diameter craniotomy was made (centered at AP: 2.0 mm posterior to bregma; ML: 3.4 mm), and the dura was removed. A double-tube system was used for tissue boring: a ceramic guide cannula (inner diameter: 1.12 mm, outer diameter: 1.48 mm, 17 gauge; Z9919P001, Kyocera) and a stainless-steel tissue cutter with a wire at the tip (inner diameter: 0.70 mm, outer diameter: 1.06 mm, 19 gauge). The cutter was rotated to create a cutting surface, and the excised tissue was removed by retracting the cutter. For neocortical recordings, a 1.5 × 1.5 mm craniotomy was made (centered at AP: 2.0 mm anterior to bregma; ML: 1.0 mm) and covered with 1.7–2.0% agar (1.5 mm thick). Whole-cell recordings were obtained from BLA neurons (AP: 1.6–3.1 mm posterior to bregma; ML: 3.0–3.7 mm; DV: 3.3–5.2 mm) or neocortical neurons (AP: 1.2–3.4 mm anterior to bregma; ML: 0.3–1.8 mm; DV: 0.8–1.7 mm) using borosilicate glass electrodes (4–7 MΩ). The intrapipette solution for current-clamp recordings contained the following (in mM): 135 K-gluconate, 4 KCl, 10 HEPES, 10 creatine phosphate, 4 MgATP, 0.3 Na_2_GTP, and 0.3 EGTA, pH 7.3, with 0.2% biocytin. For voltage-clamp recordings, the solution contained the following (in mM): 130 CsMeSO_4_, 10 CsCl, 10 HEPES, 10 creatine phosphate, 4 MgATP, 0.3 NaGTP, 10 QX-314, and 0.2% biocytin. Neurons were classified as putative excitatory (regular-spiking) or inhibitory (fast-spiking) based on firing patterns elicited by 500 ms current steps from −200 to +200 pA in 50 pA increments; only regular-spiking neurons were included in the analyses. Recordings were excluded if series resistance exceeded 75 MΩ or if the mean corrected membrane potential exceeded −50 mV. Recordings were truncated if resting potential increased by >8 mV from the initial value. For neocortical LFP recordings, a craniotomy (AP: 1.5–2.5 mm anterior to bregma; ML: 0.2 mm) was performed, and signals were recorded from the prelimbic and anterior cingulate cortices (AP: 0.4–2.5 mm anterior to bregma; ML: 0.1–1.0 mm; DV: 1.5–2.7 mm) using a tungsten electrode (UEWMGCSEKNNM, FHC) coated with DiI. Electrode placement was verified post hoc through DiI track observation. In some animals, LFPs from the olfactory bulb were recorded using a screw electrode after craniotomy (AP: 4.0 mm anterior to bregma; ML: 0.5 mm lateral). Signals from glass electrodes were amplified with a MultiClamp 700B amplifier, and tungsten and screw electrode signals were amplified with a DAM80 AC differential amplifier. All data were digitized at 20 kHz via a Digidata 1440A digitizer.

### In vitro patch-clamp recording

Mice previously injected with AAV-CAG-hChR2-H134R-tdTomato were briefly anesthetized with isoflurane prior to decapitation and brain extraction. A 400-µm-thick coronal slice containing the mPFC was prepared using a vibratome (VT1200S, Leica) at a cutting speed of 0.08 mm/s in ice-cold, oxygenated modified artificial cerebrospinal fluid (modified aCSF) containing the following (in mM): 222.1 sucrose, 27 NaHCO_3_, 1.4 NaH_2_PO_4_, 2.5 KCl, 0.5 ascorbic acid, 1 CaCl_2_, and 7 MgSO_4_. Sections were incubated at 37°C for a minimum of 30 min in oxygenated aCSF containing the following (in mM): 127 NaCl, 1.6 KCl, 1.24 KH_2_PO_4_, 1.3 MgSO_4_, 3 CaCl_2_, 26 NaHCO_3_, and 10 d-glucose. Each solution was continuously oxygenated by bubbling with a mixture of 95% O_2_/5% CO_2_. Whole-cell recordings were performed in a submerged chamber perfused with oxygenated aCSF at a flow rate of 7–9 ml/min at room temperature. Recordings were obtained from tdTomato-expressing pyramidal cells under infrared differential interference contrast microscopy. Light pulses (5 ms) at frequencies of 1, 5, 10, 20, 50, or 100 Hz were delivered using LEX2-LZ4 (473 nm, 1.5 mW, Brain Vision).

### Histology

After recordings, mice were transcardially perfused with 4% paraformaldehyde. Brains were postfixed overnight and coronally sectioned (100 µm).

For unit recording and calcium imaging experiments, sections were stained with 0.4% NeuroTrace 435/455 (N21479, Thermo Fisher Scientific) for 4 h at room temperature. To verify projection preservation, sections were permeabilized with blocking solution [phosphate-buffered saline (PBS) with 0.3% Triton X-100 and 5% bovine serum albumin (BSA; 01281-26, Nacalai Tesque)] for 1 h, incubated overnight with rabbit anti-TagRFP (1:500; R10367, Thermo Fisher Scientific) in blocking solution, and labeled with Alexa Fluor 594-conjugated secondary antibodies [1:500; Goat anti-Rabbit IgG (H+L) Highly Cross-Adsorbed Secondary Antibody, Alexa Fluor 594; A11037, Thermo Fisher Scientific] along with 0.4% NeuroTrace 435/455 in blocking solution for 1.5 h at room temperature.

For patch-clamp recordings, sections were incubated with 2 µl/ml streptavidin-Alexa Fluor 594 conjugate (S11227, Thermo Fisher Scientific) and 0.2% Triton X-100 for 4 h to identify recorded neurons. Neocortical sections were subsequently incubated with 0.4% NeuroTrace 435/455 for an additional 4 h. For BLA recordings, sections were permeabilized in blocking solution for 1 h and then incubated overnight with primary antibodies (guinea pig anti-parvalbumin, 195 004, Synaptic Systems; 1:500, and rat anti-somatostatin, MAB354, Merck Millipore; 1:500). After PBS washing, sections were incubated for 1.5 h with secondary antibodies [Goat anti-Guinea Pig IgG (H+L) Highly Cross-Adsorbed Secondary Antibody, Alexa Fluor 488, A11073, and Goat anti-Rat IgG (H+L) Cross-Adsorbed Secondary Antibody, Alexa Fluor 647, A21247, both from Thermo Fisher Scientific; 1:500 each] along with 2 µl/ml streptavidin-Alexa Fluor 594 conjugate and 0.4% NeuroTrace 435/455 in blocking solution.

In cases using thick slices and not requiring immunohistochemistry, brains were sectioned at 300–500 µm, and recorded neurons were visualized using the IsoSca*l*eSQ method (Sato et al., 2019), combined with 2 µl/ml streptavidin-Alexa Fluor 594 conjugate and 0.4% NeuroTrace 435/455. Fluorescent images were acquired using confocal microscopes (FV1000, Olympus; A1 HD25, Nikon) and merged for analysis. Recordings were excluded from analysis if virus injection sites or recording sites extended outside the BLA.

### Slow oscillation and depolarization detection

Neocortical LFPs were first band-stop filtered at respiratory frequencies (estimated from olfactory bulb LFPs via fast Fourier transform; Sato et al., 2018) and then bandpass filtered at 0.3–3 Hz. Instantaneous phase and amplitude were calculated using the Hilbert transform (Valencia et al., 2013), with LFP peaks and troughs assigned phases 0 and ±*π*, respectively. To establish an LFP-based definition of UP and DOWN states, UP-state onsets and offsets were initially identified from simultaneously recorded neocortical membrane potentials using the MAUDS algorithm (Seamari et al., 2007). The corresponding LFP phases were then extracted. The circular mean phases of UP-state onset and offset were 1.88 and −1.60 rad, respectively (Fig. S1), and these values were used as the LFP phase boundaries for UP and DOWN states. Intervals from each UP-state onset boundary to the subsequent offset boundary were defined as UP states, and the intervening intervals were defined as DOWN states. For normalized phase analyses, UP-state and DOWN-state onsets were mapped to 0 (or 2*π*) and *π*, respectively, such that each UP–DOWN cycle was represented on a 0–2*π* phase scale. BLA depolarizations were also detected using the MAUDS algorithm. For each depolarization event, the peak was defined as the time point at which the membrane potential reached its maximum value, including action-potential peaks when present.

### Unit analysis

Spike sorting was performed using Kilosort2 (Pachitariu et al., 2024) and was manually curated in Phy (https://github.com/kwikteam/phy). For chronic BLA recordings, non-REM sleep was estimated from frontal cortical LFP and neck EMG. EMG power (high-pass filtered at 100 Hz) and delta power (0.3–3 Hz) from the frontal cortical LFP were computed in 6 s windows with 5 s overlap. For each animal, the distributions of EMG and delta power were log-transformed and fit with a two-component lognormal mixture model. The minimum between the two components was used as the threshold. Time bins with EMG power below the EMG threshold and delta power above the delta threshold were classified as non-REM sleep. For spike sorting and all subsequent unit analyses of chronic BLA data, only non-REM segments were used: continuous voltage traces were first segmented into non-REM epochs, which were then concatenated and provided as input to Kilosort2. Light responsiveness was evaluated using a modified SALT method (Kvitsiani et al., 2013), by replacing Jensen–Shannon divergence with Bhattacharyya distance. For multiunit analysis, units recorded on the same channel were pooled. For single-unit analysis, only putative pyramidal cells were included based on waveform features and anatomical localization using CellExplorer (Petersen et al., 2021). Units with firing rate–amplitude correlation >0.2 or an interspike interval violation (within 3 ms) >1% were excluded.

### Cross-correlation analysis

Cross-correlations between neocortical UP state onset and neuronal activity were calculated separately for each unit, ROI, or recorded cell. The resulting cross-correlation functions were then averaged across all individually analyzed units, ROIs, and cells. Thus, the rate plots represent the mean of the individually analyzed units, ROIs, or cells, rather than rates derived from pooled data.

To quantify the relationship between UP state duration and response timing, we calculated the center-of-mass latency of the UP onset-aligned rate trace for each unit, ROI, or recorded cell and for each UP-duration group. The center of mass was defined as ΣtR(*t*)/ΣR(*t*), where *R*(*t*) is the firing rate, calcium event rate, or depolarization rate in each time bin. Unless otherwise noted, the center-of-mass latency was calculated within the corresponding UP-state window for each UP-duration group. Units, ROIs, or cells for which the center-of-mass latency could not be defined because no events were detected in the analyzed window were treated as missing for that condition.

### Calcium activity detection

Regions of interest (ROIs) were manually or algorithmically placed using ImageJ or Cellpose (Stringer et al., 2021). Background fluorescence was removed via principal component analysis. Fluorescence changes (Δ*F* / *F*) were calculated as (*F*_t_ − *F*_0_) / *F*_0_, where *F*_t_ was the fluorescence intensity at time *t*, and *F*_0_ was the lowest 5–10% of values in a ±5 s window. Signals were further denoised using a modified Okada filter (Ishikawa et al., 2020). Calcium transients were identified with an event threshold at +3 SD and onset/offset thresholds of +0.2 SD. Events were retained only if the *z*-score of the peak Δ*F* / *F* exceeded 1.96, following visual confirmation of the event. To quantify the spatial organization of calcium activity, a binary activity vector was generated for each ROI, with time points falling within detected calcium events assigned a value of 1 and all other time points assigned a value of 0. Pairwise similarity of calcium activity was quantified as the cosine similarity between the binary activity vectors for each ROI pair. Cosine similarity was calculated separately using time points belonging to UP and DOWN states. Inter-ROI distance was calculated as the Euclidean distance between ROI centroids.

### Postsynaptic conductance analysis

Excitatory and inhibitory postsynaptic conductances were calculated as *I*_E/I_ / (*V*_h_ − *E*_rev,E/I_), where *I*_E/I_ is the excitatory or inhibitory current at a given time; *V*_h_ is the holding potential (−70 mV for EPSGs, +10 mV for IPSGs); and *E*_rev,E/I_ denotes the reversal potential (0 mV for EPSGs, −90 mV for IPSGs; Funayama et al., 2016; Noguchi et al., 2022). For neuron-level comparisons between UP and DOWN states, raw conductance traces were first averaged across slow-oscillation cycles within each neuron after phase normalization, and mean conductance was then calculated separately over the entire normalized UP (0–*π*) and DOWN (*π*–2*π*) phases. The UP-minus-DOWN conductance change was calculated for each neuron. For phase-dependent analysis, the mean conductance during the DOWN state was subtracted as a baseline, and the resulting conductance traces were plotted across the normalized phase of the slow-oscillation cycle. The E/I ratio was calculated from the raw conductance values. The baseline-subtracted conductance changes were plotted in ΔEPSG-versus-ΔIPSG space to visualize phase evolution.

### Experimental design and statistical analysis

Sample sizes for each experiment are reported in the Results and figure legends and correspond to the number of animals, neurons, units, ROIs, or ROI pairs analyzed, as appropriate. Data processing and statistical analyses were performed using MATLAB (MathWorks). *p* values are reported as exact values where appropriate; values smaller than 0.0001 are reported as *p* < 0.0001. Statistical tests were selected according to data structure and distribution and included Rayleigh tests and *V* tests for circular phase distributions, permutation tests for comparison of circular mean phase, Wilcoxon rank-sum tests and Wilcoxon signed-rank tests for nonparametric comparisons, paired *t* tests where appropriate, Jonckheere–Terpstra tests for ordered trends, Friedman tests for repeated nonparametric comparisons, *χ*^2^ tests for contingency tables, and Pearson’s correlation analyses, with statistical inference based on *t* tests or cluster bootstrap confidence intervals as appropriate. Normality was assessed using the Lilliefors test. For paired comparisons, normality was assessed for the distribution of paired differences, whereas for comparisons between groups, normality was assessed separately for each group. For *V* tests, the predefined late-phase direction was set at *μ* = 3*π*/4, corresponding to the midpoint of the late phase (*π*/2 to *π*) of the UP state. In the permutation test, condition labels were randomly shuffled 10,000 times. For the EPSG–IPSG phase-profile correlation, Pearson's correlation coefficient was calculated between the population-average conductance profiles, and the 95% confidence interval was estimated using a cluster bootstrap with 10,000 resamples, in which neurons were resampled with replacement separately for EPSGs and IPSGs while retaining all cycles from each selected neuron. The study was not preregistered. Data supporting the findings of this study are available from the corresponding author upon reasonable request. Viruses generated in this study are available from the corresponding author upon reasonable request and completion of a materials transfer agreement.

### Code accessibility

Custom MATLAB code used for data preprocessing, event detection, spike analysis, and statistical analysis is available from the corresponding author upon reasonable request. The code was developed for the analyses in this study and is provided for research use.

## Results

### UP and DOWN periods are defined based on neocortical LFPs

We investigated the temporal relationship between spike activity in BLA neurons and the phases of neocortical slow oscillations by recording with silicon probes in urethane-anesthetized mice. Neocortical slow oscillations consist of alternating UP states, characterized by sustained depolarization in pyramidal cells, and DOWN states, characterized by hyperpolarization (Stern et al., 1997). These cellular dynamics are reflected in local field potentials (LFPs); in our recordings, UP states manifest as negative deflections with superimposed high-frequency activity, whereas DOWN states appear as relatively flat, slowly varying positive waveforms (Fig. S1*A*).

To determine UP and DOWN states based on LFP signals, we simultaneously recorded LFPs from the medial prefrontal cortex and whole-cell membrane potentials from pyramidal cells. Using a previously described method (Seamari et al., 2007), we identified the onsets and offsets of UP states from membrane potential traces (Fig. S1*A*, top). The simultaneously acquired LFPs were then bandpass filtered (0.3–3 Hz) to isolate slow oscillatory components (Fig. S1*A*, middle), and instantaneous phases were extracted with the Hilbert transform, with peaks defined as 0 and troughs as *π* and −*π* (Fig. S1A, bottom). UP-state onsets were significantly nonuniformly distributed across the LFP phase (Fig. S1*B*; mean phase = 1.88 rad, 95% confidence interval [CI] [1.85, 1.90], *n* = 9,668 UP-state onsets, *p* < 0.0001, *z* = 2.17 × 10^3^, Rayleigh test), and DOWN-state onsets (i.e., UP-state offsets) showed a similarly nonuniform distribution (Fig. S1C; mean phase = −1.60 rad, 95% CI [−1.62, −1.57], *n* = 9,675 DOWN-state onsets, *p* < 0.0001, *z* = 2.60 × 10^3^, Rayleigh test). These mean phases were therefore used as the LFP phase boundaries for UP-state onset and offset, respectively, in subsequent analyses.

### BLA neuronal firing is phase-locked to neocortical slow oscillations

Using silicon probes, we recorded BLA neuronal activity while simultaneously monitoring neocortical LFPs in urethane-anesthetized mice (Fig. 1*A*,*B*). BLA neurons were more active during neocortical UP states, particularly in their later phases (Fig. 1*C*). We therefore categorized UP state events into three duration groups with mean lengths of 0.50, 1.00, and 1.50 s and plotted BLA firing rates aligned to UP state onset (Fig. 1*D*). These duration-binned correlograms suggested that the latency from UP state onset to BLA firing increased with longer UP states. The distribution of UP state durations under urethane anesthesia is shown in Figure 1*E*. To quantify this relationship directly, we calculated the center-of-mass latency of the unit-wise UP onset-aligned firing-rate traces within the corresponding UP-state window for each UP-duration group. We used center-of-mass latency as a robust estimate of response timing because peak-bin estimates can be sensitive to bin-to-bin fluctuations. The center-of-mass latency shifted later as UP state duration increased (0.5 s group: 0.26 ± 0.02 s; 1.0 s group: 0.57 ± 0.10 s; 1.5 s group: 0.83 ± 0.20 s; *n* = 12 units; *p* < 0.0001, *χ*²_(2)_ = 22.17, Friedman test), providing quantitative support for a duration-dependent shift in the timing of BLA firing relative to UP-state onset.

**Figure 1.**
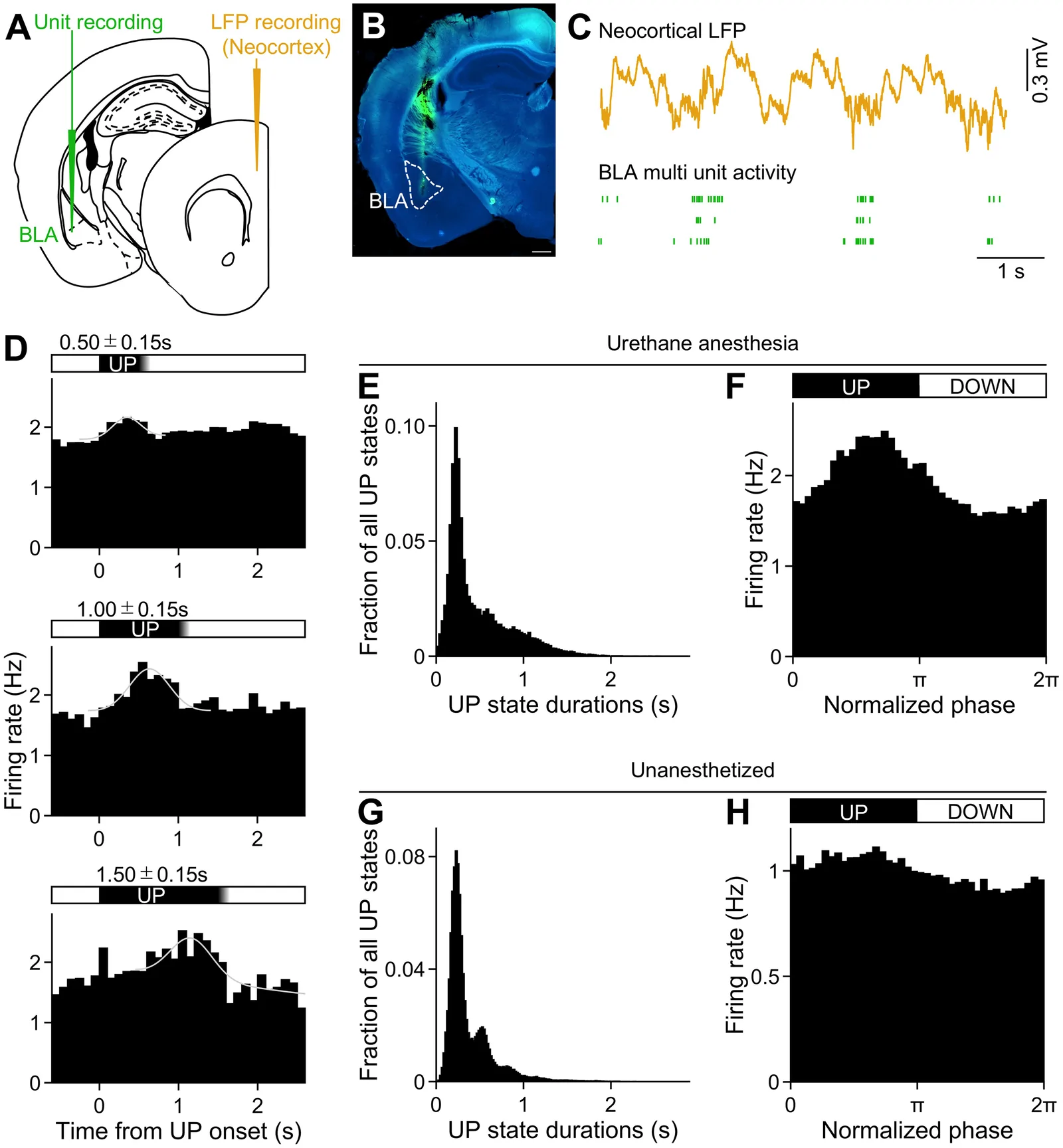
BLA unit activity is phase-locked to neocortical slow oscillations. ***A***, Schematic of unit recordings in the BLA with simultaneous LFP recording from the neocortex. ***B***, Post hoc histological confirmation of the silicon probe location in the BLA (green). Blue, Nissl staining. Scale bar, 500 µm. ***C***, Representative traces showing simultaneously recorded neocortical LFP (top) and BLA multiunit activity (bottom) under urethane anesthesia. ***D***, Cross-correlograms demonstrating the UP state duration-dependent temporal relationship between UP state onset and BLA firing. Firing rates are plotted separately for three UP state duration groups (from top to bottom), with ranges of 0.35–0.65 s, 0.85–1.15 s, and 1.35–1.65 s, resulting in mean durations of 0.50, 1.00, and 1.50 s, respectively (*n* = 12 units from 4 mice). Black, UP state; white, DOWN state. ***E***, Distribution of UP state durations under urethane anesthesia. ***F***, Histogram of BLA firing across the normalized phase of slow oscillations in urethane-anesthetized mice. *n* = 120,556 spikes from 12 units in 4 mice, *p* < 0.0001, *z* = 1,566.2, Rayleigh test; *p* < 0.0001, *V* = 1.33 × 10^4^, *V* test for the predefined late-phase direction. ***G***, Distribution of UP state durations in unanesthetized mice during non-REM sleep. ***H***, Histogram of BLA firing across the normalized phase of slow oscillations in unanesthetized mice during non-REM sleep. *n* = 209,067 spikes from 19 units in 4 mice, *p* < 0.0001, *z* = 1,048.5, Rayleigh test; *p* < 0.0001, *V* = 1.13 × 10^4^, *V* test for the predefined late-phase direction.

To investigate how BLA neuronal activity aligns with neocortical oscillations, we calculated firing rates as a function of the normalized phase of slow oscillations. This analysis revealed a significant preference during the late phase of the UP state (Fig. 1*F*; mean phase = 2.12 rad, 95% CI [2.08, 2.15], *n* = 120,556 spikes recorded from 12 units in 4 mice, *p* < 0.0001, *z* = 1,566.25, Rayleigh test; *p* < 0.0001, *V* = 1.33 × 10^4^, *V* test for the predefined late-phase direction [*µ* = 3*π*/4]).

We conducted most experiments in urethane-anesthetized mice because urethane anesthesia induces stable neocortical UP and DOWN states (Steriade et al., 1993a,b,c). To determine whether this phase preference was also present in the absence of anesthesia, we examined BLA activity in four unanesthetized mice during non-REM sleep. The distribution of UP state durations in these unanesthetized recordings is shown in Figure 1*G*. Consistent with the anesthetized condition, BLA neurons in these unanesthetized animals also exhibited a late-phase firing preference during the UP state (Fig. 1*H*; mean phase = 1.66 rad, 95% CI [1.62, 1.70], *n* = 209,067 spikes recorded from 19 units in 4 mice, *p* < 0.0001, *z* = 1,048.49, Rayleigh test; *p* < 0.0001, *V* = 1.13 × 10^4^, *V* test for the predefined late-phase direction). However, direct comparison between the two conditions revealed that the phase distributions were not identical. The mean phase was significantly shifted between anesthetized and unanesthetized mice (Δmean phase = 0.46 rad, *p* = 0.0001, permutation test on circular mean difference). In addition, unit-wise phase modulation was significantly stronger under anesthesia, as quantified by the late-UP modulation index (anesthetized: 0.204 ± 0.185, *n* = 12 units in 4 mice; unanesthetized: 0.069 ± 0.033, *n* = 19 units in 4 mice; *p* = 0.027, *z* = 2.21, Wilcoxon rank-sum test) and mean resultant vector length (anesthetized: 0.207 ± 0.146; unanesthetized: 0.077 ± 0.016; *p* = 0.0019, *z* = 3.10, Wilcoxon rank-sum test). Together, these findings indicate that BLA neurons exhibit a late-phase firing bias in both anesthetized and unanesthetized conditions, but the strength and detailed phase distribution of this bias are state dependent and more pronounced under anesthesia.

### Calcium transients in the BLA are more synchronized among nearby neurons

We examined the temporal and spatial organization of BLA neuronal activity using calcium imaging through an implanted GRIN lens (Fig. 2*A–D*). The calcium-event rate during UP states was 0.011 ± 0.005 Hz [mean ± SD of 470 regions of interest (ROIs) from 18 mice], demonstrating that BLA neurons were only sparsely active even during UP states. We next categorized UP states into three duration groups and plotted calcium-event rates aligned to UP state onset (Fig. 2*E*). Similar to the unit recording results, these duration-binned profiles suggested that calcium-event timing shifted later as UP state duration increased. This relationship was quantified by center-of-mass latency analysis of ROI-wise UP onset-aligned calcium-event-rate traces across UP-duration groups (0.5 s group: 0.25 ± 0.11 s; 1.0 s group: 0.49 ± 0.19 s; 1.5 s group: 0.71 ± 0.34 s; *n* = 71 ROIs; *p* < 0.0001, *χ*^2^_(2)_ = 79.30, Friedman test). To examine the phase distribution across individual ROIs, we plotted ROI-wise calcium-event profiles across the normalized phase of slow oscillations together with their average event-rate profile (Fig. 2*F*). Calcium events were preferentially associated with the UP state and showed a significant bias toward its later phase (mean phase = 1.68 rad, 95% CI [1.61, 1.75], *n* = 4,891 events from 470 ROIs in 18 mice, *p* < 0.0001, *z* = 336.50, Rayleigh test; *p* < 0.0001, *V* = 990.30, *V* test for the predefined late-phase direction).

**Figure 2.**
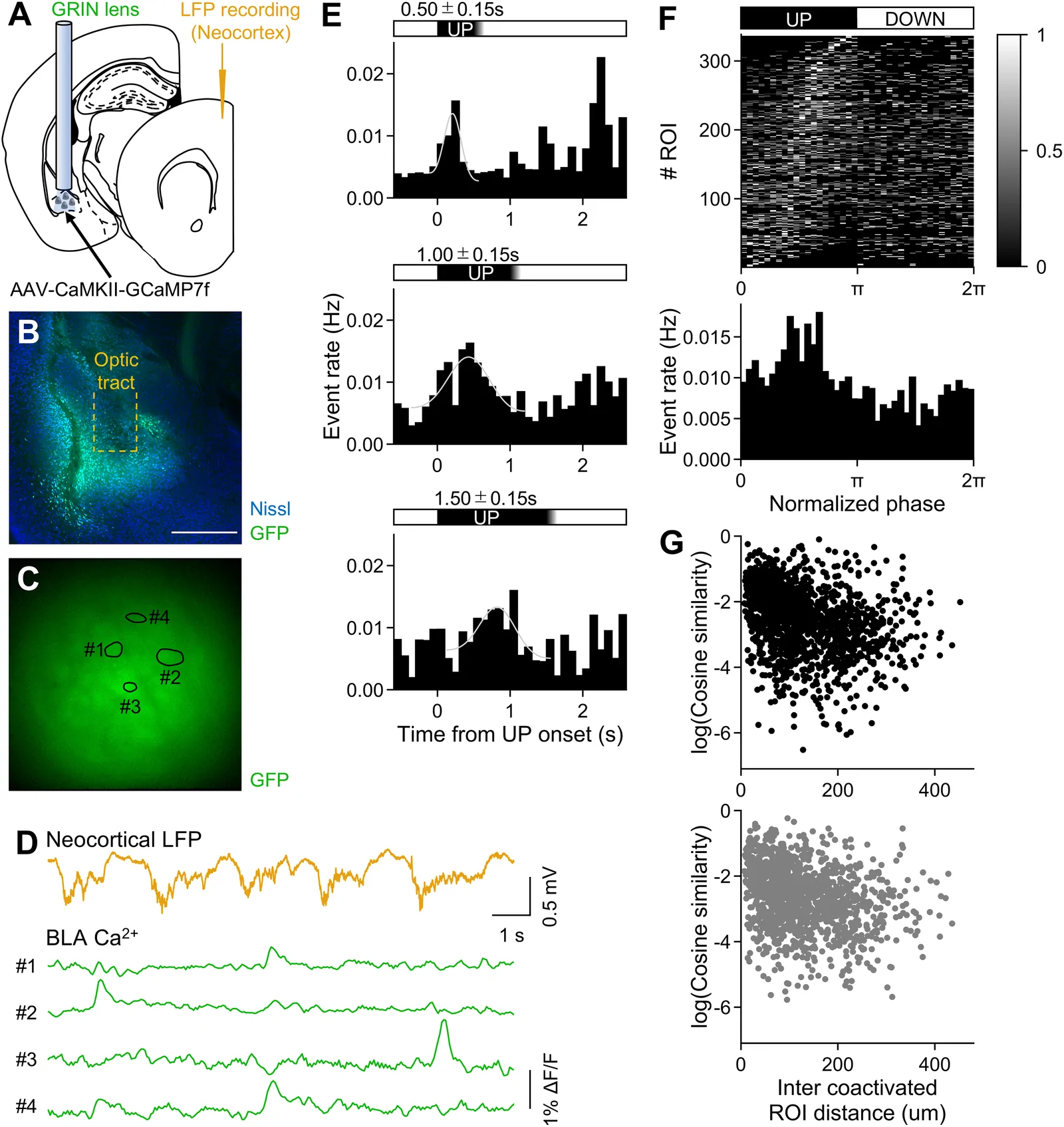
Calcium activity of BLA neurons is phase-locked to neocortical UP states. ***A***, Schematic of GRIN lens implantation and viral expression strategy targeting the BLA, with simultaneous neocortical LFP recording. ***B***, Post hoc histological verification of GCaMP7f expression in the BLA and visualization of the optic tract. Green, GFP; blue, Nissl. Scale bar, 500 µm. ***C***, Representative field of view for calcium imaging in the BLA. ***D***, Simultaneously recorded traces of neocortical LFP (top) and Ca^2+^ signals from four representative BLA ROIs (bottom) under urethane anesthesia. ***E***, Same as Figure 1*D*, but for calcium event onsets. Event rates are plotted separately for three UP state duration groups, with ranges of 0.35–0.65 s, 0.85–1.15 s, and 1.35–1.65 s (*n* = 470 ROIs from 18 mice). Black, UP state; white, DOWN state. ***F***, ROI-wise calcium-event profiles across the normalized phase of slow oscillations (top) and corresponding mean event-rate profile (bottom). Each row in the heatmap represents an individual ROI, with event rates normalized within each ROI for visualization. ROIs are ordered according to the center of mass of their calcium-event profiles during the UP states. *n* = 4,891 events from 470 ROIs in 18 mice, *p* < 0.0001, *z* = 336.50, Rayleigh test; *p* < 0.0001, *V* = 990.30, *V* test for the predefined late-phase direction. ***G***, Spatial organization of temporally synchronized BLA activity during UP (top) and DOWN (bottom) states. Cosine similarity of calcium activity between ROI pairs was negatively correlated with inter-ROI distance in both states. UP: *n* = 1,567 ROI pairs. *R* = −0.31, *p* < 0.0001, *t*_(1565)_ = −12.97; DOWN: *n* = 1,250 ROI pairs. *R* = −0.28, *p* < 0.0001, *t*_(1248)_ = −10.33; *t* tests of correlation coefficient.

We next examined the spatial organization of BLA calcium activity by relating pairwise cosine similarity of calcium activity to inter-ROI distance. Significant negative correlations were observed during both UP and DOWN states (Fig. 2*G*; UP: *n* = 1,567 ROI pairs, *R* = −0.31, *p* < 0.0001, *t*_(1565)_ = −12.97; DOWN: *n* = 1,250 ROI pairs, *R* = −0.28, *p* < 0.0001, *t*_(1248)_ = −10.33, *t* tests of correlation coefficients). Thus, this distance-dependent synchronization was not unique to UP states but rather reflected a general local spatial organization of BLA calcium activity. These findings indicate that BLA calcium activity is sparse during slow oscillations, with calcium events biased toward the later phase of neocortical UP states and greater synchronization among nearby neurons.

### A tissue-boring method enables in vivo whole-cell recordings from the BLA

To investigate the membrane mechanisms underlying the late-phase bias of BLA neuronal activity, we addressed a significant technical hurdle: in vivo whole-cell patch-clamp recordings from deep brain regions like the BLA are notoriously challenging, despite being necessary for directly measuring subthreshold membrane dynamics.

To overcome this hurdle, we developed a novel tissue-boring method to facilitate reliable in vivo whole-cell recordings from the BLA (Fig. 3*A*). This method uses a concentric double-tube system, consisting of a guide cannula for the patch pipette and an inner tissue cutter, to create a stable access path. The cutter, equipped with a wire at its tip, is rotated to create a cutting surface at the bottom of the cannula track. Subsequent retraction of the cutter removes the excised tissue, thereby creating a physical path for pipette insertion.

**Figure 3.**
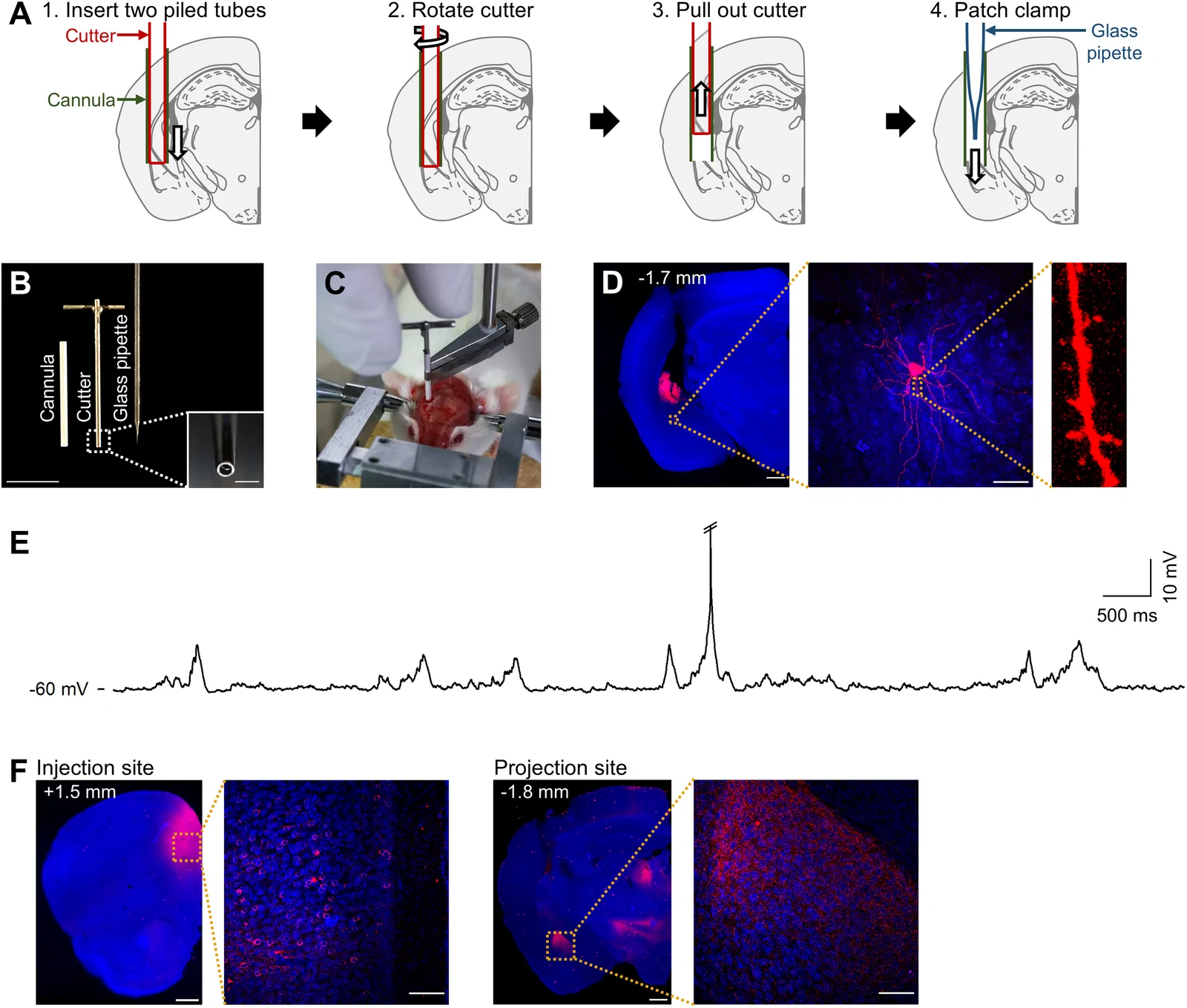
A novel tissue-boring method enables reliable in vivo whole-cell recording from the BLA. ***A***, Schematic illustration of the tissue-boring procedure for deep brain access. ***B***, Photograph of the double-tube components used in the method: ceramic guide cannula (left), stainless-steel tissue cutter (middle), and a glass pipette (right). Inset, magnified view of the tissue cutter tip showing the cutting wire. Scale bars, 1 cm in the low-magnification image and 2 mm in the inset image. ***C***, Photograph of the surgical setup showing the stacked guide cannula and tissue cutter inserted into the brain, with rotation applied to the cutter for tissue removal. ***D***, Post hoc visualization of a patch-clamped BLA neuron. The high-magnification image on the right confirms excitatory identity via spine detection. Blue, Nissl staining; red, biocytin. Scale bars, 500 µm in the low-magnification images and 50 µm in the enlarged images. ***E***, Representative whole-cell recording trace showing membrane potential fluctuations in a BLA neuron. ***F***, Verification of mPFC→BLA projections following the tissue-boring procedure in separate viral tracing experiments. Left, AAV_DJ_-hSyn-Synaptophysin-mCherry injection site in the mPFC. Right, labeled axonal fibers in the BLA. Blue, Nissl staining; red, mCherry. Scale bars, 500 µm in the low-magnification images and 100 µm in the enlarged images. *n* = 6 mice.

To implement this design (Fig. 3*B*,*C*), we constructed the system using a ceramic guide cannula (inner *Φ*: 1.12 mm; outer *Φ*: 1.48 mm; 17 gauge) and a stainless-steel tissue cutter (inner *Φ*: 0.70 mm; outer *Φ*: 1.06 mm; 19 gauge). The diameters were carefully selected to ensure stable access for standard patch pipettes (outer *Φ*: ∼1 mm) while minimizing damage to surrounding brain tissue. This approach allowed us to obtain whole-cell recordings from BLA neurons and subsequently perform histological visualization of the recorded neurons (Fig. 3*D*,*E*).

A potential concern with this technique is that the tissue-boring procedure could inadvertently damage axonal fibers connecting the BLA with other brain regions. To address this concern, we performed separate viral tracing experiments by injecting a viral tracer into the medial prefrontal cortex (mPFC), a principal upstream region of the BLA. In sections close to the recording level along the anterior-posterior axis, mPFC axonal projections to the BLA were still observed following the tissue-boring process (Fig. 3*F*; AP −1.8 mm; recording example in Fig. 3*D*, AP −1.7 mm).

### BLA neuronal depolarizations are modulated by the phase and duration of neocortical UP states

We investigated the relationship between BLA membrane potential dynamics and neocortical slow oscillations. Our whole-cell recordings demonstrated that BLA neurons underwent depolarizations during neocortical UP states, although these events were sparse and did not occur on every cycle (Fig. 4*A*). Thus, neocortical UP states did not deterministically trigger BLA depolarizations. Instead, they appeared to provide a temporal window in which BLA depolarizations could occur. Crucially, depolarizations typically emerged during the later phase of the UP state, suggesting that their timing might vary with UP-state duration.

**Figure 4.**
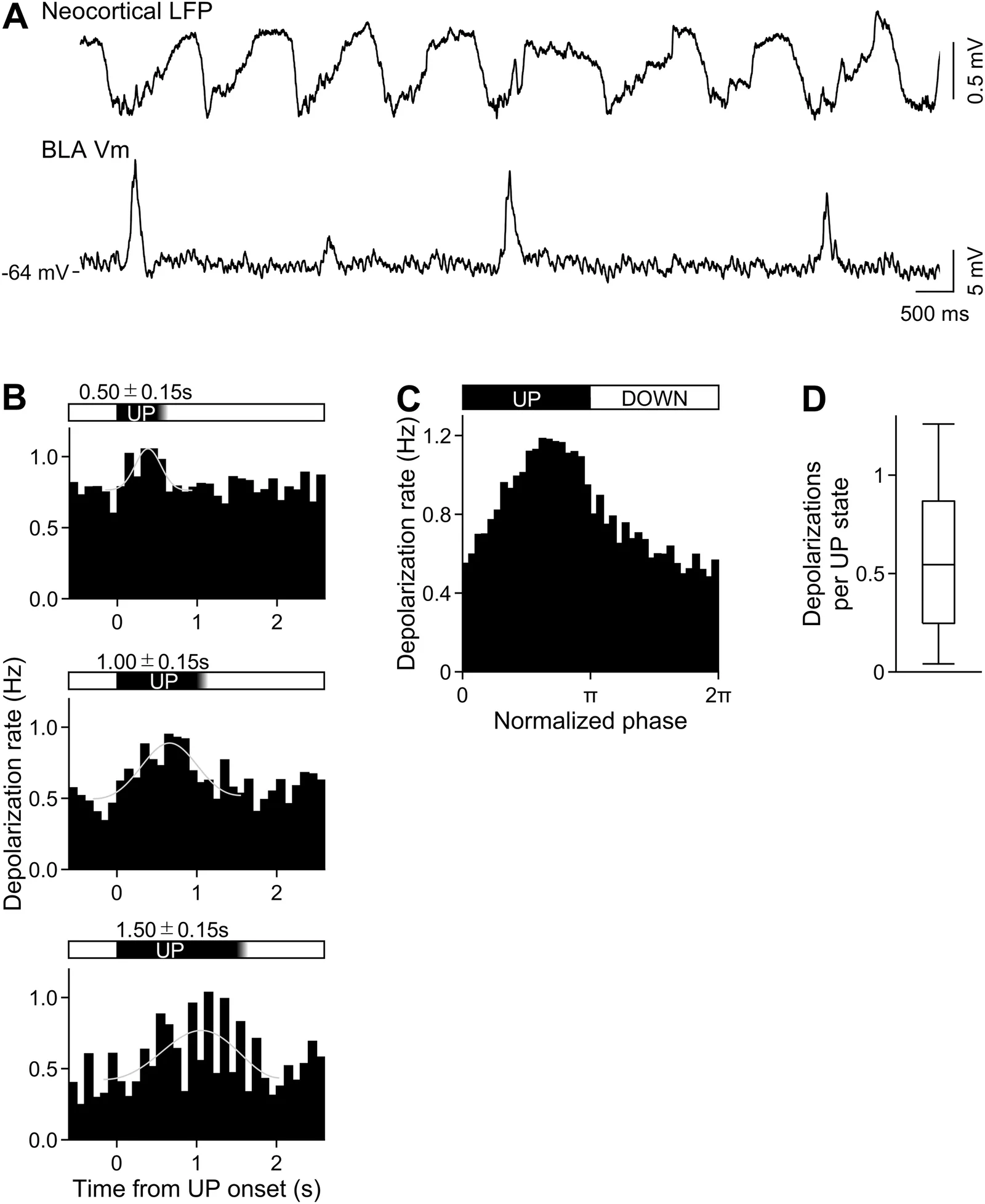
BLA neuronal depolarizations are phase-locked to neocortical UP states. ***A***, Representative traces showing simultaneously recorded neocortical LFP (top) and membrane potential of a BLA neuron (bottom) under urethane anesthesia. ***B***, Same as Figure 1*D*, but for the peak time of BLA depolarization. Depolarization rates are plotted separately for three UP state duration groups, with ranges of 0.35–0.65 s, 0.85–1.15 s, and 1.35–1.65 s (*n* = 27 neurons). Black, UP state; white, DOWN state. ***C***, Histogram of depolarization peaks relative to the normalized phase of slow oscillations, showing a bias toward the late phase. *n* = 15,980 depolarizations from 27 neurons, *p* < 0.0001, *z* = 358.19, Rayleigh test; *p* < 0.0001, *V* = 2.33 × 10^3^, *V* test for the predefined late-phase direction. ***D***, Box plot showing the number of BLA depolarizations per UP state across all recorded neurons (*n* = 27).

To test this hypothesis, we calculated the rates of BLA depolarizations as a function of UP state duration (Fig. 4*B*). These duration-binned traces suggested that the latency from UP state onset to BLA depolarization increased with longer UP states. We further quantified this relationship using center-of-mass latency analysis of cell-wise UP onset-aligned depolarization rate traces. The center-of-mass latency increased with UP state duration (0.5 s group: 0.27 ± 0.04 s; 1.0 s group: 0.54 ± 0.11 s; 1.5 s group: 0.81 ± 0.16 s; *n* = 21 cells; *p* < 0.0001, *χ*^2^_(2)_ = 40.10, Friedman test), supporting a duration-dependent shift in depolarization timing.

We next quantified depolarization rates relative to the normalized phase of the UP state. The resulting phase distribution was significantly nonuniform, exhibiting a bias toward the late phase (Fig. 4*C*; mean phase = 2.58 rad, 95% CI [2.51, 2.66], *n* = 15,980 depolarizations from 27 neurons, *p* < 0.0001, *z* = 358.19, Rayleigh test; *p* < 0.0001, *V* = 2.33 × 10^3^, *V* test for the predefined late-phase direction). Despite their phase bias, BLA depolarizations remained sparse, as indicated by the low number of depolarizations per UP state (Fig. 4*D*; *n* = 27 neurons).

To further evaluate the relationship between UP state duration and BLA depolarization, we calculated the proportion of UP states in which a given BLA neuron was depolarized, grouped by UP state length. We observed that the proportion of UP states accompanied by BLA depolarizations increased with longer UP states (Fig. S2*A*; *n* = 27 neurons [0–0.5 s, 0.5–1 s], *n* = 24 neurons [1–1.5 s], *n* = 21 neurons [1.5–2 s], *n* = 6 neurons [>2 s], *p* < 0.0001, *z* = 6.92, Jonckheere–Terpstra test).

We then investigated the temporal pattern of UP states accompanied by BLA depolarization. A purely random process would predict that the distribution of interevent intervals (i.e., the number of intervening UP states without BLA depolarization) would approximate the geometric distribution. The observed interevent interval rates differed markedly across interval lengths, with the highest rate occurring for consecutive UP states with no intervening UP state (Fig. S2B; *p* < 0.0001, *χ*^2^_(20)_ = 387.76, Friedman test). To directly test whether consecutive occurrence exceeded that expected under independence, we compared the probability of BLA depolarization occurring in two consecutive UP states, *P*(with*_t_*_-1_∩with*_t_*), with the probability expected under independence, *P*(with)^2^. The observed probability was significantly higher than the expected probability (Fig. S2C, left; *n* = 27 neurons, *p* < 0.0001, *t*_(26)_ = 4.85, paired *t* test). Similarly, the probability of no depolarization occurring in two consecutive UP states, *P*(without*_t_*_-1_∩without*_t_*), was significantly higher than *P*(without)^2^ (Fig. S2C, right; *n* = 27 neurons, *p* = 1.10 × 10^−4^, *z* = 3.87, Wilcoxon signed-rank test). These findings suggest that BLA depolarizations preferentially occur in consecutive UP states. Thus, the sparse occurrence of BLA depolarizations was not random but was structured by UP state duration and recent UP state history. Further analysis revealed that the normalized phase of BLA depolarization peak shifted later as the UP state duration increased (Fig. S2D; *n* = 2,208 depolarizations [0–0.5 s], *n* = 3,788 depolarizations [0.5–1 s], *n* = 2,193 depolarizations [1–1.5 s], *n* = 423 depolarizations [1.5–2 s], *n* = 44 depolarizations [>2 s], *p* < 0.0001, *z* = 9.32, Jonckheere–Terpstra test). Together, these analyses indicate that neocortical UP states provide a probabilistic temporal window for BLA depolarization, with depolarization probability depending on UP-state duration and recent UP-state history and depolarization timing varying with UP-state duration.

### Membrane potential dynamics in the BLA differ from those in the neocortex

A comparison of the membrane potential dynamics of BLA and prefrontal cortical neurons under urethane anesthesia revealed several key differences between the two regions (Fig. 5*A*). Because cortical activity under this condition was dominated by slow oscillations, most of the observed membrane potential fluctuations were associated with neocortical slow oscillations. First, the frequency of depolarizations in BLA neurons did not differ significantly from that of neocortical UP states (Fig. 5*B*; neocortex: 0.64 ± 0.20 Hz, *n* = 16 neurons; BLA: 0.85 ± 0.50 Hz, *n* = 32 neurons; mean ± SD, *p* = 0.16, *z* = 1.41, Wilcoxon rank-sum test). Second, BLA neuronal depolarizations exhibited a significantly shorter duration than the neocortical UP states observed in neocortical neurons (Fig. 5*C*; neocortex: 0.82 ± 0.28 s, *n* = 16 neurons; BLA: 0.40 ± 0.20 s, *n* = 32 neurons; mean ± SD, *p* < 0.0001, *z* = −4.47, Wilcoxon rank-sum test). Third, BLA neurons exhibited a significantly lower number of spikes per depolarization event compared with neocortical counterparts (Fig. 5*D*; neocortex: 1.96 ± 2.27 spikes, *n* = 11 neurons; BLA: 0.17 ± 0.29 spikes, *n* = 15 neurons; mean ± SD, *p* = 0.022, *z* = −2.28, Wilcoxon rank-sum test). Fourth, the proportion of depolarizations containing at least one spike relative to the total number of depolarizations was significantly lower in the BLA than in the neocortex (Fig. 5*E*; neocortex: 45.11 ± 37.63%, *n* = 11 neurons; BLA: 6.97 ± 8.88%, *n* = 15 neurons; mean ± SD, *p* = 0.011, *z* = −2.54, Wilcoxon rank-sum test). Collectively, these results demonstrate that BLA neuronal depolarizations are briefer and less frequently associated with spiking than neocortical UP states during slow oscillations. These observed disparities suggest that distinct intrinsic or synaptic mechanisms shape the unique properties of BLA membrane dynamics.

**Figure 5.**
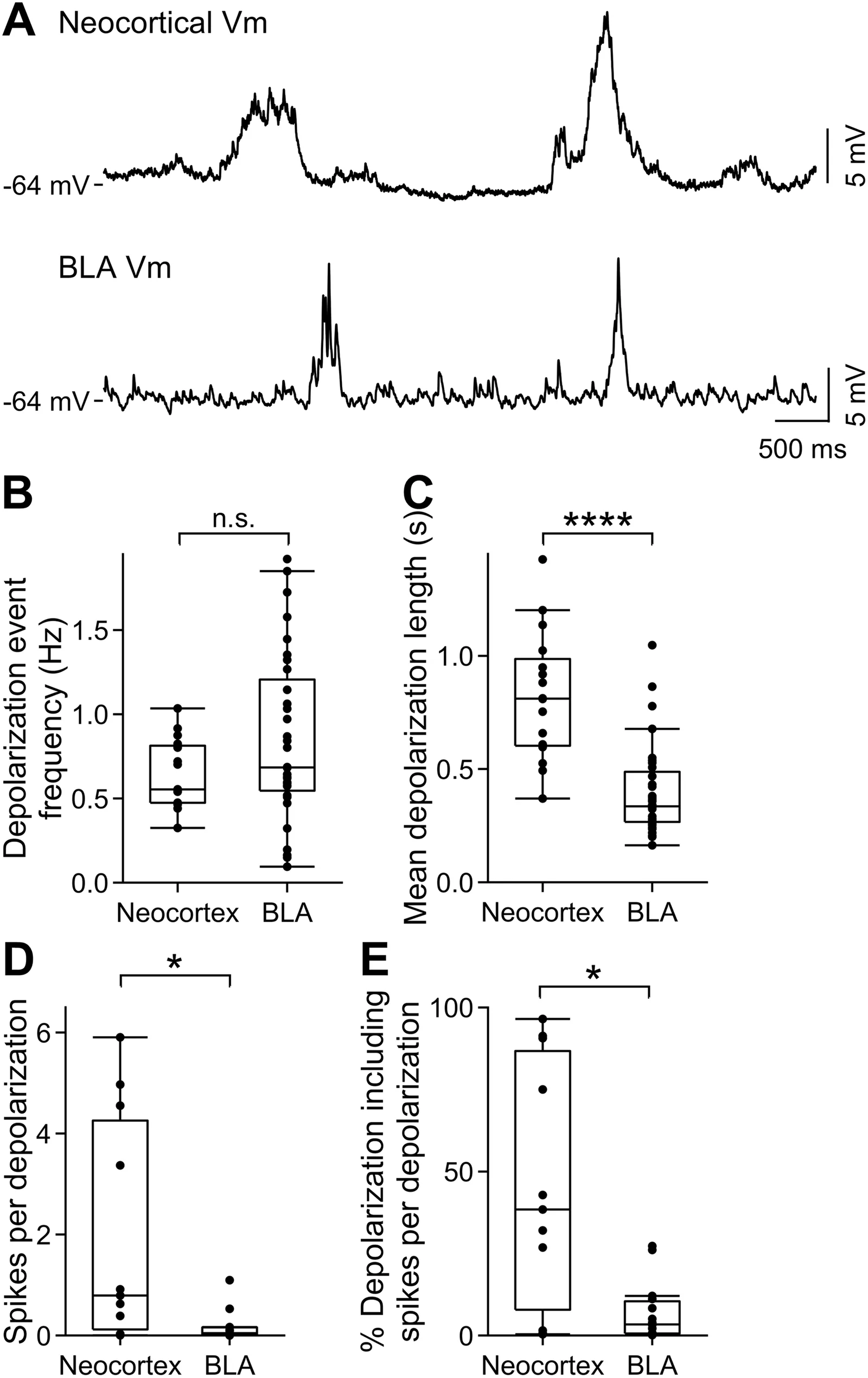
Membrane potential dynamics differ between neocortical and BLA neurons. ***A***, Representative traces of simultaneously recorded membrane potentials from a neocortical neuron (top) and a BLA neuron (bottom) under urethane anesthesia. ***B***, Comparison of depolarization event frequency between neocortical and BLA neurons. Neocortex: *n* = 16 neurons; BLA: *n* = 32 neurons. *p* = 0.16, *z* = 1.41, Wilcoxon rank-sum test. ***C***, Comparison of depolarization duration. Neocortex: *n* = 16 neurons; BLA: *n* = 32 neurons, ****p < 0.0001, *z* = −4.47, Wilcoxon rank-sum test. ***D***, Comparison of spikes per depolarization event. Neocortex: *n* = 11 neurons; BLA: *n* = 15 neurons, *p = 0.022, *z* = −2.28, Wilcoxon rank-sum test. ***E***, Comparison of the proportion of depolarizations containing at least one spike. Neocortex: *n* = 11 neurons; BLA: *n* = 15 neurons, *p = 0.011, *z* = −2.54, Wilcoxon rank-sum test. ***D*** and ***E*** include only neurons that fired at least one spike during the recording period, whereas ***B*** and ***C*** include all recorded neurons.

### Postsynaptic conductances differ between BLA and prefrontal neurons

To investigate the mechanisms underlying the distinct membrane potential dynamics observed in the BLA compared with the neocortex, we measured excitatory and inhibitory postsynaptic conductances (EPSGs and IPSGs) during neocortical slow oscillations. Neurons were voltage clamped at holding potentials of −70 mV and +10 mV to isolate EPSGs and IPSGs, respectively, using a cesium-based internal solution (Fig. 6*A*).

**Figure 6.**
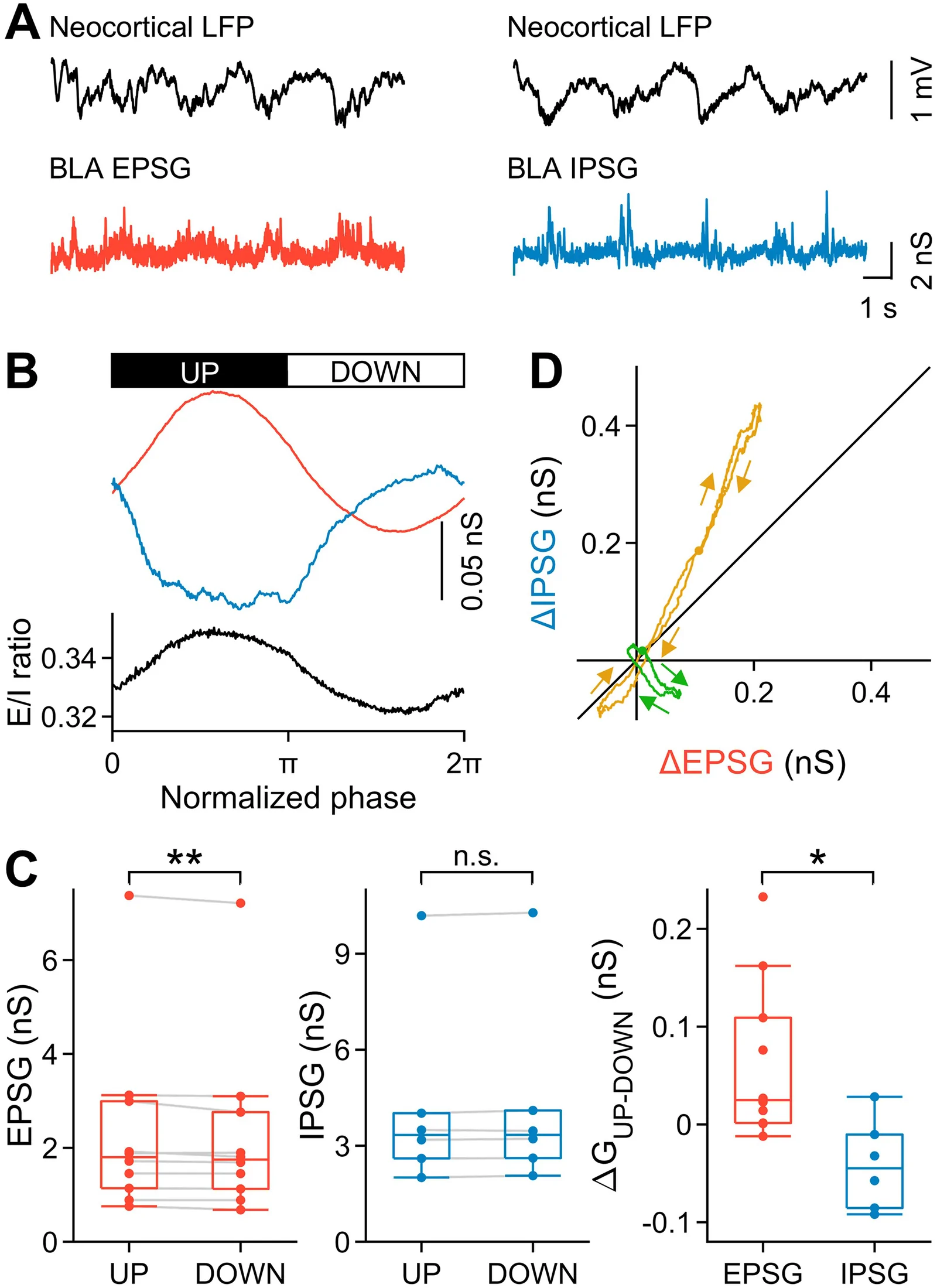
Postsynaptic conductance dynamics in BLA neurons during slow oscillations differ from those in neocortical neurons. ***A***, Representative traces showing neocortical LFP (top) and BLA postsynaptic conductances (bottom), recorded under urethane anesthesia. Left, excitatory postsynaptic conductance (EPSG); right, inhibitory postsynaptic conductance (IPSG). ***B***, Top, Time-averaged traces of EPSG (red) and IPSG (blue) across the normalized phase of slow oscillations, calculated from 2,280 and 935 cycles recorded from 10 and 6 BLA neurons, respectively. The population-average EPSG and IPSG phase profiles were negatively correlated across the normalized UP–DOWN cycle (Pearson’s *r* = −0.923, neuron-level cluster bootstrap 95% CI [−0.974, −0.655]). Bottom, Ratio of EPSG to IPSG across the oscillation phase. ***C***, Individual-neuron conductance measurements. Left, Paired mean EPSG values during the UP and DOWN states (*n* = 10 neurons, **p = 0.0098, Wilcoxon signed-rank test). Middle, Paired mean IPSG values during the UP and DOWN states (*n* = 6 neurons, *p* = 0.094, Wilcoxon signed-rank test). Right, UP-minus-DOWN conductance changes for EPSG recordings (*n* = 10 neurons) and IPSG recordings (*n* = 6 neurons; *p = 0.011, Wilcoxon rank-sum test). ***D***, Phase evolution of baseline-subtracted synaptic conductance changes plotted in ΔEPSG–ΔIPSG space. The trajectory (green, BLA; yellow, neocortex) highlights the anticorrelated E/I relationship in the BLA, contrasting with the neocortical balance. Arrows represent the transition from UP state onset to DOWN state offset. Dots indicate the UP state onset.

We analyzed phase-dependent changes in EPSGs and IPSGs across 2,280 and 935 slow-oscillation cycles, obtained from 10 and 6 neurons, respectively (Fig. 6*B*). The conductance traces plotted across the normalized phase showed that EPSG and IPSG changes were temporally anticorrelated across the UP–DOWN cycle (Fig. 6*B*, top; Pearson's *r* = −0.923, neuron-level cluster bootstrap 95% CI [−0.974, −0.655]). EPSG increased during the UP phase and declined during the DOWN phase, whereas IPSG showed the opposite pattern, decreasing during the UP phase and increasing during the DOWN phase. To quantify these changes at the neuron level, we compared mean raw conductance across the entire normalized UP and DOWN phases for each neuron. EPSG was significantly higher during UP than during DOWN states (Fig. 6*C*, left; UP: 2.33 ± 1.94 nS; DOWN: 2.26 ± 1.90 nS; mean ± SD, *n* = 10 neurons, *p* = 0.0098, Wilcoxon signed-rank test), whereas IPSG showed the opposite direction of modulation, although this difference did not reach statistical significance (Fig. 6*C*, middle; UP: 4.25 ± 2.99 nS; DOWN: 4.29 ± 3.02 nS; mean ± SD, *n* = 6 neurons, *p* = 0.094, Wilcoxon signed-rank test). Furthermore, the UP-minus-DOWN conductance change differed significantly between EPSGs and IPSGs (Fig. 6*C*, right; EPSG: 0.063 ± 0.081 nS, *n* = 10 neurons; IPSG: −0.042 ± 0.046 nS, *n* = 6 neurons; mean ± SD, *p* = 0.011, Wilcoxon rank-sum test), with EPSGs and IPSGs showing shifts in opposite directions. Consistent with this anticorrelated pattern, the E/I ratio increased during the UP phase and decreased during the DOWN phase (Fig. 6*B*, bottom).

We then plotted the temporal evolution of baseline-subtracted conductance changes in ΔEPSG-versus-ΔIPSG space across oscillation cycles (Fig. 6*D*, green line). Notably, changes in EPSGs and IPSGs in the BLA were anticorrelated throughout the oscillation cycle. This conductance trajectory was oriented approximately orthogonally to that observed in neocortical neurons, which typically show balanced excitatory and inhibitory inputs (Fig. 6*D*, yellow line), consistent with prior reports (Shu et al., 2003; Haider et al., 2006).

These findings suggest that the distinct membrane dynamics of BLA neurons during slow oscillations may reflect a distinct organization of synaptic input, characterized by an anticorrelated relationship between excitation and inhibition. This E/I configuration contrasts with the E/I balance observed in the neocortex and may contribute to the sparse and phase-biased depolarization patterns observed in the BLA.

### BLA-projecting mPFC neurons sustain firing into the late phase of UP states

Finally, we investigated whether BLA-projecting mPFC neurons exhibit a temporal firing pattern that could contribute to the late-phase bias of BLA activity during neocortical slow oscillations. To identify mPFC neurons that project to the BLA, we employed an optogenetic tagging strategy using a retrograde viral vector (AAV-CAG-hChR2-H134R-tdTomato) injected into the BLA (Fig. 7*A–C*). In vitro whole-cell recordings confirmed that channelrhodopsin-2 (ChR2)-expressing neurons in the mPFC responded reliably to 5 Hz photostimulation but not to 10 Hz stimulation (Fig. S3). This in vitro validation supported the use of 5 Hz photostimulation for opto-tagging. We applied 5 Hz photostimulation to the mPFC during in vivo recordings to identify light-responsive mPFC neurons as BLA-projecting (Fig. 7*D*).

**Figure 7.**
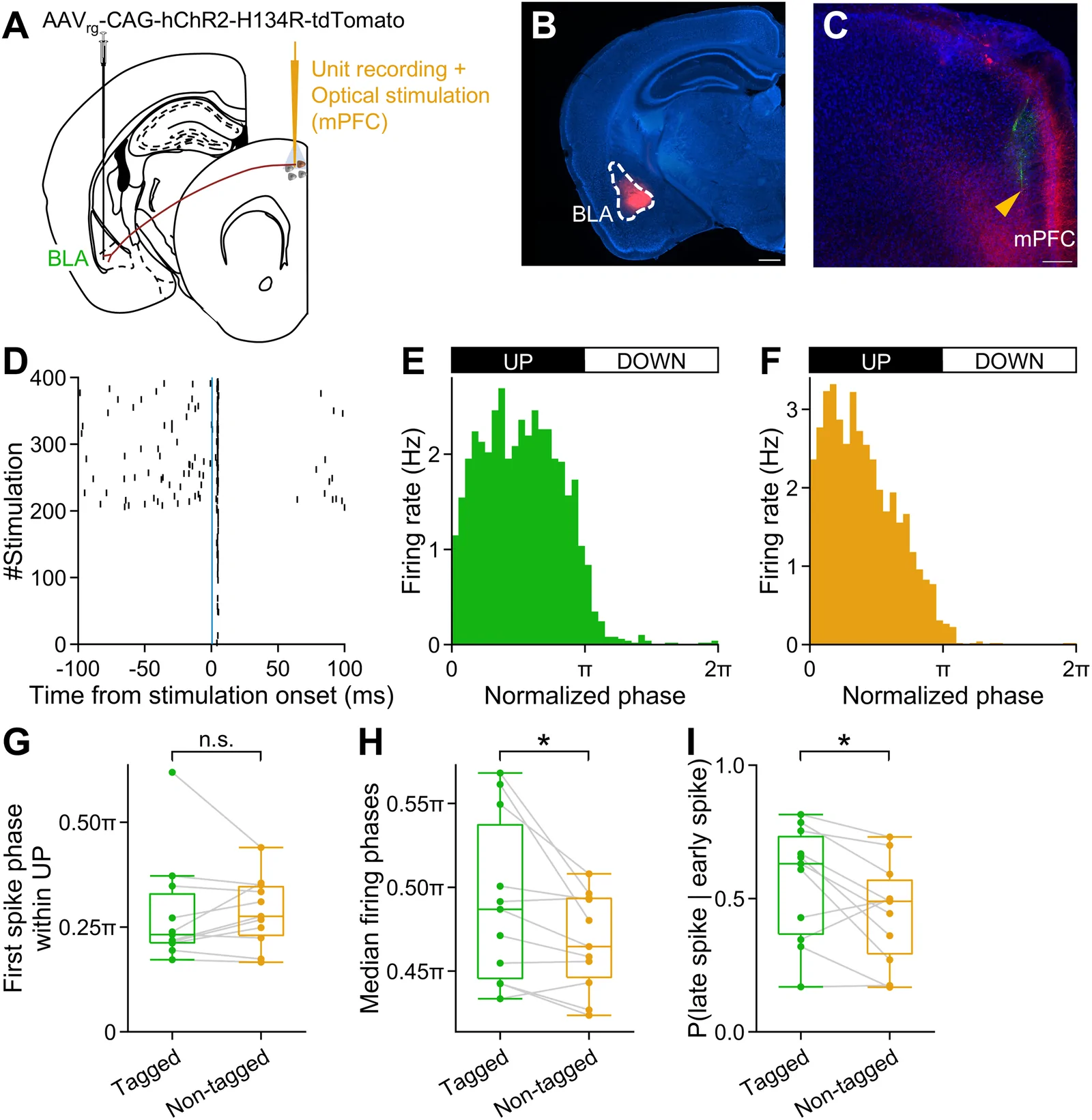
BLA-projecting mPFC neurons sustain firing into the late phase of neocortical UP states. ***A***, Schematic showing unit recording and optical stimulation in the mPFC, with retrograde labeling of BLA-projecting neurons via AAV injection into the BLA. ***B***, Histological confirmation of the virus injection site in the BLA. Red, tdTomato; blue, Nissl staining. Scale bar, 500 µm. ***C***, Retrogradely labeled mPFC neurons expressing tdTomato (red), with a silicon probe shank visible (green). The orange arrowhead indicates the shank tip. Blue, Nissl staining. Scale bar, 200 µm. ***D***, Spike raster plot from a representative opto-tagged mPFC unit aligned to light-stimulation onset during 5 Hz photostimulation. Blue shading indicates the light-stimulation period. ***E***, Spike phase distribution of an opto-tagged (light-responsive) mPFC neuron relative to the phase of slow oscillations. ***F***, Spike phase distribution for a non-tagged (non-light-responsive) mPFC neuron. ***G***, Comparison of the first-spike phase within UP states between tagged and non-tagged units across animals. The first-spike phase did not differ significantly between groups (*n* = 11 mice, *p* = 0.58, Wilcoxon signed-rank test). ***H***, Comparison of median firing phases across animals between tagged and non-tagged units (*n* = 11 mice, *p = 0.025, *t*_(10)_ = 2.63, paired *t* test). ***I***, Probability of firing during the late phase of an UP state conditional on firing during its early phase, compared between opto-tagged and non-tagged units across animals (*n* = 11 mice, *p = 0.029, *t*_(10)_ = 2.54, paired *t* test).

We then analyzed the firing phases of opto-tagged (BLA-projecting) and non-tagged mPFC neurons relative to neocortical slow oscillations. Opto-tagged neurons preferentially fired during the late phase of the UP state (Fig. 7*E*), whereas non-tagged neurons tended to fire earlier (Fig. 7*F*).

To determine whether this difference reflected delayed recruitment of BLA-projecting mPFC neurons, we first compared the phase of the first spike within individual UP states. The first-spike phase did not differ significantly between opto-tagged and non-tagged units across animals (Fig. 7*G*; opto-tagged: 0.88 ± 0.40 rad; non-tagged: 0.90 ± 0.26 rad; mean ± SD, *n* = 11 mice, *p* = 0.58, Wilcoxon signed-rank test), indicating that BLA-projecting mPFC neurons were not recruited later within the UP state. In contrast, despite the similar first-spike phase, the overall median firing phase of opto-tagged units was significantly later than that of non-tagged units across animals (Fig. 7*H*; opto-tagged: 1.54 ± 0.15 rad; non-tagged: 1.47 ± 0.09 rad; mean ± SD, *n* = 11 mice, *p* = 0.025, *t*_(10)_ = 2.63, paired *t* test). This dissociation suggested that the later overall firing phase of opto-tagged units might reflect a greater likelihood of continuing to fire into the late phase rather than delayed recruitment. Consistent with this interpretation, opto-tagged units showed a significantly higher probability of firing during the late phase of an UP state if they had fired during its early phase (Fig. 7*I*; opto-tagged: 0.56 ± 0.21; non-tagged: 0.45 ± 0.19; mean ± SD, *n* = 11 mice; *p* = 0.029, *t*_(10)_ = 2.54, paired *t* test).

Together, these findings indicate that the late-phase firing preference of BLA-projecting mPFC neurons is not explained by delayed recruitment but is more consistent with a greater likelihood of continuing to fire into the late phase of UP states. These results suggest that BLA-projecting mPFC neurons represent a potential source of temporally biased cortical input that may contribute to the late-phase bias of BLA depolarizations.

## Discussion

We demonstrate four physiological features of neocortex–BLA coordination during slow oscillations. First, BLA neurons rarely fire during slow oscillations, but when they do, their activity is biased toward the late phase of the neocortical UP state, a pattern evident in both spiking and subthreshold depolarization. This late-phase preference was observed in both anesthetized and unanesthetized conditions, although the strength and detailed phase distribution of this bias were state dependent, with a stronger bias under anesthesia. Second, BLA membrane potential dynamics are associated with an anticorrelated E/I configuration. Third, BLA neuronal activity shows local spatial organization, with proximal neurons exhibiting more strongly synchronized activity. Fourth, BLA-projecting mPFC neurons are more likely than non-tagged neurons to continue firing into the late phase of the UP state. These results provide novel insights into the physiological architecture of neocortex–BLA coordination during slow oscillations.

We found that both the timing and probability of BLA depolarizations varied with the duration of neocortical UP states. Specifically, longer UP states were associated with a greater likelihood of BLA depolarization and with depolarizations occurring later within the UP state. This duration dependence is particularly relevant given that individual cortical UP states exhibit considerable variability in duration (Jercog et al., 2017). More broadly, slow cortical activity spans a range of frequencies and temporal scales (Norton and Jewett, 1965; Penttonen and Buzsáki, 2003). Our findings extend these observations by demonstrating that variability in UP-state duration is associated with systematic changes in both the likelihood and timing of BLA depolarizations. Importantly, the sparse occurrence of BLA depolarizations indicates that neocortical UP states do not deterministically trigger BLA activity. Rather, UP states appear to provide a probabilistic temporal window for BLA depolarization, such that both its probability and timing vary with UP-state duration and recent UP-state history. This probabilistic coupling may reflect a requirement for additional afferent inputs or permissive local circuit states within the BLA.

We observed marked differences in membrane potential dynamics between BLA and neocortical neurons during slow oscillations. Compared with neocortical UP states, BLA depolarizations were consistently shorter, contained significantly fewer spikes, and were significantly less likely to contain action potentials. These physiological distinctions were associated with a distinct temporal organization of synaptic conductances. Indeed, voltage-clamp recordings revealed that changes in EPSGs and IPSGs in the BLA were temporally anticorrelated across the slow-oscillation cycle, in contrast to the balanced E/I patterns observed in the neocortex (Compte et al., 2003; Haider et al., 2006). This anticorrelated E/I pattern may contribute to the sparse and temporally biased activity of BLA neurons.

The observation that BLA-projecting mPFC neurons are more likely than non-tagged neurons to remain active during the late phase of UP states suggests that they may provide temporally biased cortical input to the BLA and could thereby contribute to the late-phase bias of BLA depolarizations. This finding complements previous studies demonstrating frequency-specific mPFC–BLA coordination across different behavioral contexts (Likhtik et al., 2014; Stujenske et al., 2014; Karalis et al., 2016; Taub et al., 2018).

The mechanisms by which BLA-projecting mPFC neurons sustain activity into the late phase of the UP state remain unclear. Neocortical neurons differ in their projection targets and molecular identities (Xu et al., 2024), raising the possibility that BLA-projecting mPFC neurons possess distinct intrinsic or synaptic properties that promote sustained firing during UP states. Beyond mPFC input, other afferent pathways may also shape the temporal organization of BLA activity. One candidate is hippocampal sharp wave-ripple (SPW-R) activity, which is temporally coordinated with neocortical slow oscillations and tends to occur near UP–DOWN state transitions (Mizuno and Ikegaya, 2024; Swanson et al., 2025). Previous studies have also demonstrated coordinated activity between the hippocampus and the BLA, including coactivation during SPW-Rs (Girardeau et al., 2017) and high-frequency synchronization between these regions (Cox et al., 2020; Miyawaki and Mizuseki, 2022). Given the widespread influence of SPW-R-associated activity across brain regions (Logothetis et al., 2012), hippocampal SPW-R activity may represent an additional temporally structured influence on the timing of BLA neuronal activity during slow oscillations.

BLA calcium activity also showed local spatial organization, with nearby neurons exhibiting more strongly synchronized activity. Previous studies have shown that genetically and projection-defined BLA neuronal populations exhibit spatial organization, ranging from the spatial segregation of genetically defined populations to intermingled projection-defined populations arranged along topographical gradients (Kim et al., 2016; Beyeler et al., 2018). Accordingly, the local synchronization observed here may be related to the known spatial heterogeneity of BLA neuronal populations.

Direct investigation of membrane potential dynamics in the BLA (Crane et al., 2009; Windels et al., 2010, 2016) remains technically challenging because in vivo whole-cell recordings from deep brain regions are difficult to perform (Margrie et al., 2002). Consequently, many mechanistic studies of BLA cellular and synaptic physiology have relied on ex vivo slice preparations (Felix-Ortiz et al., 2013; Hübner et al., 2014; Senn et al., 2014; Namburi et al., 2015; Xu et al., 2016; Kasugai et al., 2019), which lack intact long-range synaptic connectivity. While alternative in vivo approaches, such as robotic obstruction-detection (Stoy et al., 2017) and tissue aspiration (Funayama et al., 2016; Matsumoto et al., 2016; Noguchi et al., 2022), have been developed, our novel tissue-boring technique offers key advantages. Specifically, this method uses a narrow-diameter cannula to minimize tissue damage and does not require custom machinery. Furthermore, the incorporated guide cannula stabilizes access to the recording site and reduces obstruction by tissue or blood (Brecht and Sakmann, 2002; Kuo and Wu, 2012). Crucially, our anatomical tracing experiments demonstrated that mPFC→BLA projections were preserved after the tissue-boring procedure.

Several limitations should be considered when interpreting these findings. First, most experiments were performed under urethane anesthesia, and the late-phase bias of BLA activity was more pronounced in this condition than in the unanesthetized condition. Moreover, the unanesthetized recordings were obtained under passive conditions. Thus, the extent to which the temporal organization described here generalizes across natural brain states and active behavioral contexts remains to be determined. Second, we did not examine these dynamics during learning or emotion-related behavioral paradigms, nor did we manipulate late-UP BLA or mPFC activity to assess their behavioral consequences. Therefore, the present findings should be interpreted as characterizing the physiological organization of neocortex–BLA coordination during slow oscillatory states rather than demonstrating a specific role in emotional or mnemonic processing. Third, although BLA-projecting mPFC neurons were more likely than non-tagged neurons to remain active into the late phase of UP states, our data do not establish a causal relationship between mPFC activity and the timing of BLA activity, nor do they determine the extent to which the temporal profile of BLA activity is inherited from cortical input, generated by intrinsic BLA mechanisms, or shaped by other afferent pathways. Finally, because the molecular identities and projection targets of the imaged BLA neurons were not determined, the relationship between the observed local synchronization and specific BLA neuronal subpopulations remains unresolved.

In conclusion, our findings demonstrate that the phase and duration of neocortical UP states are closely associated with both the probability and timing of BLA neuronal activity, revealing a temporally structured, probabilistic mode of neocortex–BLA coordination during slow oscillations. These findings provide new physiological insights into the organization of BLA activity relative to neocortical slow oscillations and identify BLA-projecting mPFC neurons as a potential source of temporally biased cortical input. Future studies using phase-specific manipulations of mPFC→BLA activity will be essential to determine the causal contribution and behavioral relevance of these temporal dynamics.
